# The genome-scale DNA-binding profile of BarR, a β-alanine responsive transcription factor in the archaeon *Sulfolobus acidocaldarius*

**DOI:** 10.1186/s12864-016-2890-0

**Published:** 2016-08-08

**Authors:** Han Liu, Kun Wang, Ann-Christin Lindås, Eveline Peeters

**Affiliations:** 1 Department of Bio-Engineering Sciences, Research Group of Microbiology, Vrije Universiteit Brussel, Pleinlaan 2, B-1050 Brussels, Belgium; 2 Department of Molecular Biosciences, The Wenner-Gren Institute, Stockholm University, Svante Arrhenius v. 20C, SE-10691 Stockholm, Sweden

**Keywords:** β-alanine, *Sulfolobus*, Leucine-responsive regulatory protein, Transcription regulation, ChIP-seq

## Abstract

**Background:**

The Leucine-responsive Regulatory Protein (Lrp) family is a widespread family of regulatory transcription factors in prokaryotes. BarR is an Lrp-like transcription factor in the model archaeon *Sulfolobus acidocaldarius* that activates the expression of a β-alanine aminotransferase gene, which is involved in β-alanine degradation. In contrast to classical Lrp-like transcription factors, BarR is not responsive to any of the α-amino acids but interacts specifically with β-alanine. Besides the juxtaposed β-alanine aminotransferase gene, other regulatory targets of BarR have not yet been identified although β-alanine is the precursor of coenzyme A and thus an important central metabolite. The aim of this study is to extend the knowledge of the DNA-binding characteristics of BarR and of its corresponding regulon from a local to a genome-wide perspective.

**Results:**

We characterized the genome-wide binding profile of BarR using chromatin immunoprecipation combined with high-throughput sequencing (ChIP-seq). This revealed 21 genomic binding loci. High-enrichment binding regions were validated to interact with purified BarR protein in vitro using electrophoretic mobility shift assays and almost all targets were also shown to harbour a conserved semi-palindromic binding motif. Only a small subset of enriched genomic sites are located in intergenic regions at a relative short distance to a promoter, and qRT-PCR analysis demonstrated that only one additional operon is under activation of BarR, namely the glutamine synthase operon. The latter is also a target of other Lrp-like transcription factors. Detailed inspection of the BarR ChIP-seq profile at the β-alanine aminotransferase promoter region in combination with binding motif predictions indicate that the operator structure is more complicated than previously anticipated, consisting of multiple (major and auxiliary) operators.

**Conclusions:**

BarR has a limited regulon, and includes also glutamine synthase genes besides the previously characterized β-alanine aminotransferase. Regulation of glutamine synthase is suggestive of a link between β-alanine and α-amino acid metabolism in *S. acidocaldarius*. Furthermore, this work reveals that the BarR regulon overlaps with that of other Lrp-like regulators.

**Electronic supplementary material:**

The online version of this article (doi:10.1186/s12864-016-2890-0) contains supplementary material, which is available to authorized users.

## Background

Microorganisms sense and respond to environmental perturbations by a variety of gene regulatory mechanisms, of which regulation by transcription factors is a major mechanism. Archaea, which constitute a prokaryotic phylogenetic domain distinct from Bacteria, are characterized by a eukaryotic-like basal transcription machinery and bacterial-like regulatory transcription factors [1]. Recently, genome-wide chromatin immunoprecipitation (ChIP) approaches prove to be a powerful methodology to map the binding profiles of regulatory transcription factors in archaeal model organisms such as *Sulfolobus* spp. and *Halobacterium salinarum* NRC1, thereby unravelling the regulon and physiological role of these factors [2–9].

One of the most abundant and best characterized regulatory transcription factor family in archaea is the bacterial/archaeal Leucine-responsive Regulatory Protein (Lrp) family [10], also known as the feast/famine regulatory protein (FFRP) family [11]. The Lrp family is an ancient family of transcription factors, of which it can be assumed that a prototype was already present in the last common ancestor of Bacteria and Archaea [12]. While the presence of *lrp*-type genes is restricted to only half of the bacterial genomes [12], all archaeal genomes are predicted to harbour *lrp*-type genes with an average of five paralogs per genome [8] suggesting that the expansion of Lrp-like transcription factors is an archaea-specific evolutionary mechanism for adaptation to environmental and nutritional changes [8]. This is corroborated by the observation that bacterial Lrp-like transcription factors are unambiguously responsive to proteinogenic amino acids, which are precursors to proteins, and are involved in the regulation of amino acid metabolism and transport, whereas some archaeal Lrp-like transcription factors have more versatile functions. These functions can encompass the regulation of energy and central metabolism or the response to oxidative stress [8, 13–17], and in these cases the Lrp proteins interact with non-proteinogenic amino acids or non-amino acid small molecule ligands [11, 17].

Members of the Lrp family consist of approximately 150 amino acids and are characterized by an amino-terminal helix-turn-helix (HTH) DNA binding domain and a carboxy-terminal αβ sandwich oligomerization and ligand response domain, called “Regulation of Amino acid Metabolism” (RAM) domain, which are connected by a flexible linker region [10, 12]. Lrp-like regulators are known to act as repressors, activators or as dual-function transcription factors [10, 18].

Just like their bacterial counterparts, archaeal Lrp-family transcription factors are either local regulators, involved in the regulation of a limited number of adjacently located genes [6, 19] and/or operons or global regulators, having an extensive regulon [14, 19]. Previously, genome-wide ChIP approaches have been used to investigate the in vivo binding profiles of archaeal Lrp-like transcription factors [6–8]. Interestingly, in two phylogenetically unrelated archaeal organisms, *Sulfolobus solfataricus* and *Halobacterium salinarum*, a large overlap was observed in the DNA-binding locations of two or more Lrp-like transcription factors [6, 8], demonstrating that they co-associate on the genome. This can be explained either by the formation of hetero-oligomeric structures, as was previously observed for *Pyrococcus* Lrp-like transcription factors [11, 20], or by the similarity in the DNA-binding motifs of the different paralogs enabling them to bind to the same sequence [8, 21].

BarR is an Lrp-type transcription factor in *Sulfolobus acidocaldarius* with a non-proteinogenic amino acid ligand [17]. Indeed, this transcription factor is specifically responsive to β-alanine, the precursor of coenzyme A (CoA), and its gene is located in a divergent operon with a gene predicted to encode β-alanine aminotransferase, an enzyme that catalyzes the first step in β-alanine degradation [17]. Deletion of the *barR* gene resulted in a decreased exponential growth rate in the presence of exogenous β-alanine in the growth medium. Furthermore, gene expression analysis demonstrated that BarR activates the expression of the adjacent aminotransferase gene, but only upon β-alanine supplementation. In contrast, BarR auto-activates the expression of its own gene in a β-alanine independent manner. Heterologously produced BarR protein displays an octameric state in solution and forms a single nucleoprotein complex by interacting with multiple sites in the 170-bp long intergenic region separating the *barR* and aminotransferase gene. In vitro, DNA binding is specifically responsive to β-alanine upon direct interaction of this amino acid with the ligand-binding pocket [17]. Intriguingly, β-alanine does not influence DNA binding of BarR in vivo.

Previously, we have focused only on the study of local interactions and regulatory effects of BarR in the genomic neighbourhood of its own gene [17]. Here, we extend the study of the physiological function of BarR to a genome-wide level. Additional direct regulatory targets of BarR are identified by chromatin immunoprecipitation combined with high-throughput sequencing (ChIP-seq) and by expression analysis of genes located in the neighbourhood of genomic binding targets. Comparative analysis with previously published genome-wide binding profiles of other Lrp-type transcription factors in *Sulfolobus* spp. demonstrate that there is an overlap in the regulon of BarR and of other Lrp-like factors.

## Methods

### Strains and growth conditions

*Sulfolobus acidocaldarius* was cultured while continuously shaking at 75 °C in Brock medium [22] supplemented with 0.2 % casamino acids, 0.2 % sucrose, 0.02 mg/ml uracil and if mentioned, with 10 mM β-alanine. While for ChIP-seq analysis *S. acidocaldarius* DSM639 was used, *S. acidocaldarius* MW001 [23] and MW001Δ*barR* [17] were grown for relative gene expression quantification experiments.

### ChIP-seq analysis

ChIP-seq analysis was performed for biological duplicates. *S. acidocaldarius* DSM639 was cultured while continuously shaking at 75 °C in Brock medium supplemented with 0.2 % casamino acids, 0.2 % sucrose, 0.02 mg/ml uracil and if mentioned, with 10 mM β-alanine. Crosslinked and sheared DNA was prepared from 200 ml cultures grown until reaching an optical density (OD_600 nm_) of 0.3 as described before [4] with the following exception: while in previously established ChIP protocols with hyperthermophilic *Sulfolobus* spp. cells were cooled down before performing formaldehyde crosslinking at a constant temperature of 37 °C, here crosslinking was performed immediately after the culture was taken out of the incubator at 75 °C [Additional file 1]. After sonication, the sizes of sheared DNA fragments ranged from less than 100 bp to about 800 bp with a major proportion of the fragments between 100 bp and 600 bp.

ChIP was performed with polyclonal anti-BarR rabbit antibodies, which were previously validated for specificity [17], using Dynabeads M-280 sheep anti-rabbit IgG beads (Life Technologies) as described previously [24]. As a control, we also prepared an antibody-free mock sample. Subsequently, all samples were purified with the iPure DNA extraction kit (Diagenode) following 1 x 50 bp sequencing with the Illumina Miseq system (Scilife Lab, Stockholm, Sweden). Sequence reads were mapped to the *S. acidocaldarius* DSM 639 genome (NC_007181.1) with Burrows-Wheeler Aligner (BWA 0.7.10) [25] with default settings and MACS version 2 (2.1.0) [26] was used for peak calling (q value cutoff = 1.00e^−8^), followed by a manual curation. ChIP-seq results were visualized by IGV version 2.3.2 [27]. DNA sequences from ChIP-seq peak regions were extracted by BED Tools [28]. All raw sequence data files are available as supporting data. Binding motifs were identified in ChIP-seq enriched regions with MEME software version 4.10.0 using default parameters [29].

### Electrophoretic mobility shift assays

Recombinant His-tagged BarR was overexpressed in *Escherichia coli* and purified from inclusion bodies as described before [17]. Electrophoretic mobility shift assays (EMSAs) were performed with DNA fragments generated by PCR using oligonucleotides [Additional file 2] of which one is 5’-end labeled with ^32^P and using *S. acidocaldarius* genomic DNA as a template. Probes were designed to harbour the predicted binding motif. EMSAs were performed as described previously [30]. All binding reactions contained an excess non-specific competitor DNA and, when indicated, 1 mM β-alanine.

### Reverse transcriptase quantitative PCR

Total RNA was isolated from exponentially growing *S. acidocaldarius* MW001 and *S. acidocaldarius* MW001Δ*barR* cultures using an RNeasy mini kit (Qiagen). Residual genomic DNA was removed by treatment with Turbo DNase (Ambion) according to manufacturer’s instructions. First-strand cDNA was synthesized from 1 μg RNA with a SuperScript III First-Strand Synthesis SuperMix kit (Invitrogen). Primers [Additional file 2] were designed with Primer3 Plus software [31]. Reverse transcriptase quantitative PCR (RT-qPCR) was carried out in a Bio-Rad iCycler as described before [6]. Biological quadruplicates were assayed and normalization was performed with respect to the expression of reference genes *Saci_0691* (encoding RNA polymerase subunit A) and *Saci_1336* (encoding TATA binding protein). A paired *t*-test was performed to validate differential expression.

## Results and discussion

### Genome-scale identification of BarR binding regions

**Fig. 1:**
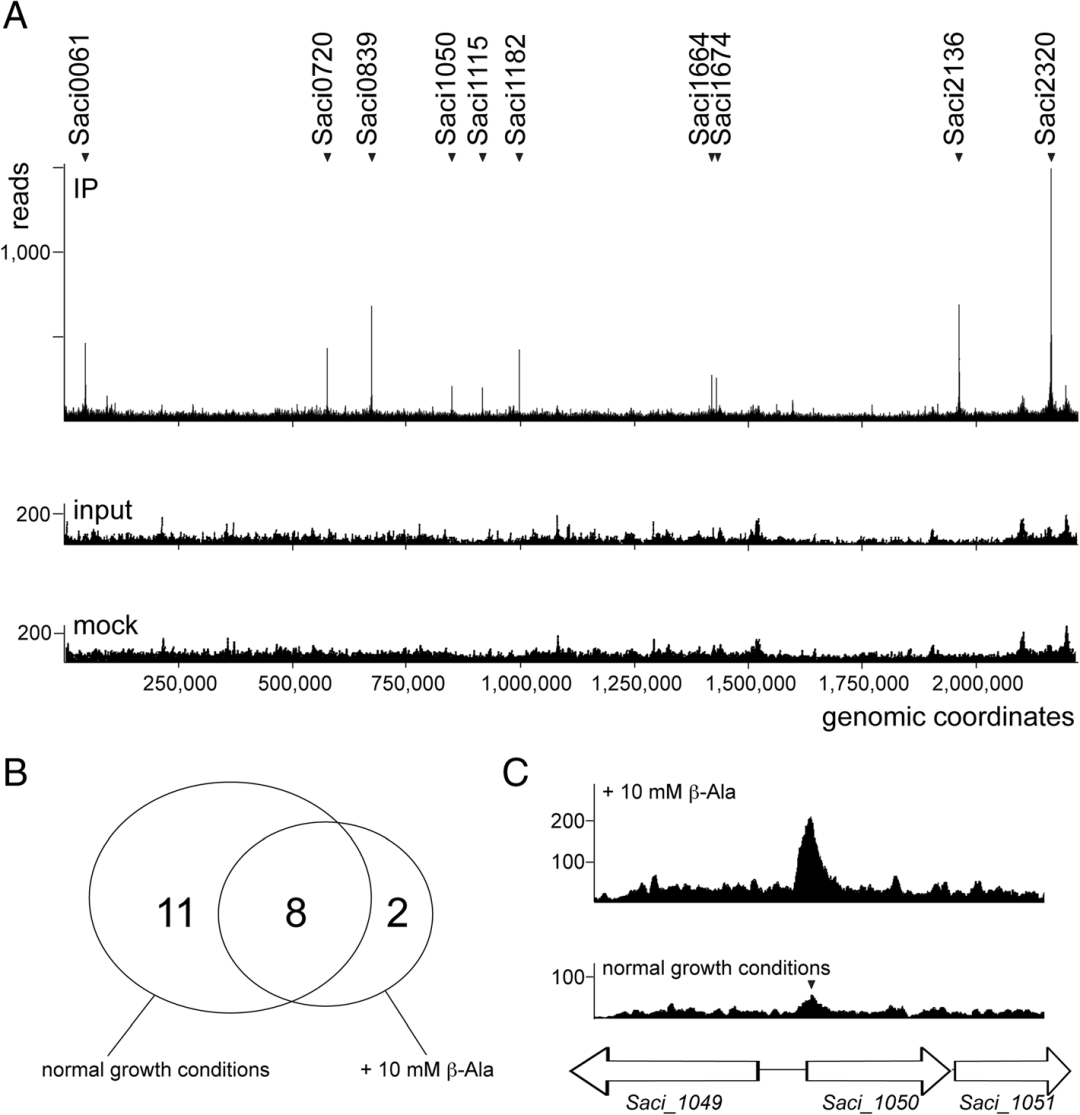
Genome-wide binding profile of the BarR regulator. **a** Overview of the BarR binding profile as determined by chromatin immunoprecipitation and sequencing (ChIP-seq) analysis. This binding profile was recorded upon supplementation of 10 mM β-alanine and resembles the profile in normal growth conditions. High-enrichment targets are indicated. **b** Venn diagram depicting the overlap in called ChIP-seq binding locations detected in the absence and presence of β-alanine. **c** An example of a binding profile (target Saci1050) displaying sequence reads recorded in the absence and presence of β-alanine. The binding peak was only called for the cells grown in the presence of β-alanine but a minor binding peak below threshold level is also visible upon growth in normal conditions (indicated with a triangle)

To obtain a genome-wide view of the in vivo DNA interactions of BarR, we performed a ChIP-seq analysis using polyclonal anti-BarR antibodies. Since β-alanine is the specific ligand of BarR, this analysis was done for cells grown in the absence but also in the presence of exogenously added 10 mM β-alanine. Sequencing libraries were constructed of input DNA and immunoprecipitated DNA of cells grown in each of these conditions, which were subjected to next-generation sequencing and mapped to the *S. acidocaldarius* genome. Upon sequencing the input samples, no obvious bias was observed with the read count evenly distributed across the genome (Fig. 1a). Furthermore, relatively low background signals were observed for a mock IP control.

Peak calling yielded in total 21 ChIP-seq peaks that represent BarR binding loci, which are distributed uniformly across the entire genome (Fig. 1a, Table 1). Eight ChIP-seq peaks were called in both growth conditions, while 11 peaks were called only upon growth without exogenously added β-alanine and 2 only upon growth in the presence of β-alanine in the culture medium (Fig. 1b, Table 1). However, for those that were detected only in one of the two conditions, below-threshold binding signals were also observed in the other condition (Fig. 1c), and thus, both profiles are similar to each other. This observation suggests that ligand binding does not strongly affect the interaction with DNA in vivo to achieve regulation as for most other canonical ligand-responding prokaryotic transcription factors. Previously, it was indeed observed that BarR performs a ligand-dependent activation of the β-alanine aminotransferase gene *Saci_2137* [17], but that ligand binding does not abrogate protein-DNA complexes in vivo. This was also demonstrated for another Lrp-type transcription factor in *S. acidocaldarius*, the lysine-dependent activator LysM [7].

**Table 1:** Locations of BarR genomic binding regions as determined by ChIP-seq

Peak summit coordinate	Growth condition^a^	Fold enrich-ment^b^	Nearest ORF	Annotation	Peak summit location^c^	*In vitro* binding^d^	Predicted binding motif	Gene regulation^e^
19904	w/o	N.A./5.3	*Saci_0028*	Hypothetical protein	intra-5’	N.A.	-	N.A.
46668	both	4.9/8.7	*Saci_0061*	Lysine arginine ornithine transport system kinase	intra-3’	++	+	-
554848	w/o	N.A./4.4	*Saci_0695*	Translation initiation factor IF-2	intra	N.A.	+	N.A.
577211	both	4.8/12.7	*Saci_0720*	Nicotinamide-nucleotide adenylyltransferase	intra-5’ (operon)	+	+	N.A.
674867	both	11.1/13.8	*Saci_0839*	Reverse gyrase	intra	++	+	-
676039	w/o	N.A./4.3	*Saci_0839*	Reverse gyrase	intra	N.A.	+	N.A.
850922	w	4.2/N.A.	*Saci_1050*	ParA, chromosome partitioning ATPase	intra-5’	+	+	-
918486	both	3.6/5.4	*Saci_1115*	Alcohol dehydrogenase	intra	+	+	N.A.
999186	both	4.0/11.9	*Saci_1182*	Major facilitator superfamily permease	intra	N.A.	+	N.A.
1421788	both	3.0/7.3	*Saci_1664*	Protein kinase	intra	N.A.	+	N.A.
1431591	w/o	N.A./5.5	*Saci_1674*	Gluconolactonase	intra-5’	++	+	-
1563444	w/o	N.A./3.7	*Saci_1796*	Hypothetical protein	intra-3’	N.A.	+	N.A.
1563826	w/o	N.A./9.2	*Saci_1796*	Hypothetical protein	inter-3’	++	+	-
1598826	w	3.9/N.A.	*Saci_1833*	Hypothetical protein	intra-3’	N.A.	+	N.A.
1773917	w/o	N.A./5.8	*Saci_1964*	Hypothetical protein	intra-5’	N.A.	+	N.A.
1840150	w/o	N.A./4.4	*Saci_2025*	ATPase	inter-3’	N.A.	+	N.A.
1889870	w/o	N.A./6.4	*Saci_2073*	Major facilitator superfamily permease	inter-3’	N.A.	+	N.A.
1964012	w/o	N.A./3.6	*Saci_2136*	BarR	intra-3’	+*	+	+*
1964490	both	4.3/6.6	*Saci_2137*	Aminotransferase	inter-5’	+*	+	+*
2000304	w/o	N.A./3.7	*Saci_2166*	Uroporphyrin-III methyltransferase	intra-5’	N.A.	+	N.A.
2166455	both	2.2/7.2	*Saci_2319*	Hypothetical protein	inter	++	+	+**

^a^Growth condition in which the binding region was detected above the significance level: both = detected in absence and presence of exogenously added β-alanine, w = detected only in the presence of β-alanine, w/o = detected only in the absence of β-alanine, ^b^fold-enrichment value in the presence of β-alanine/in the absence of β-alanine against input sample, ^c^location of the peak summit with respect to the nearest gene: intra = intragenic, inter = intergenic, 5’ = within 150 bp (intragenic) or 300 bp (intergenic) of the 5’ end of the gene, 3’ = within 150 bp (intragenic) or 300 bp (intergenic) of the 3’ end of the gene. “Operon” means that the gene for which the peak summit is located within 150 bp of the 5’ end, is a second or next gene that is part of an operon, ^d^EMSA results, with “+” indicating the observation of binding and “++” indicating the observation of high-affinity binding, ^e^qRT-PCR results. N.A. = not analyzed; an asterisk indicates that this result was previously published [17]; a double asterisk indicates that it is not the nearest ORF that is regulated. All gene annotations were manually curated

The largest fraction of the BarR binding loci are exclusively located in intragenic regions and moreover, besides the *barR*/*Saci_2137* intergenic region, only a limited number of binding loci that encompass an intergenic region are located at a reasonably short distance from promoter regions to potentially cause regulation of transcription initiation (i.e., Saci0028, Saci1050, Saci1674, Saci1964 and Saci2166 peaks). Genes located in the direct neighbourhood of the peaks have a variety of functions, including amino acid metabolism (lysine arginine ornithine transport system, *gltB*) and sugar metabolism (gluconolactonase). Our analysis did not reveal binding in the neighbourhood of a malonate semialdehyde dehydrogenase gene (*Saci_1700*), which catalyzes the second step in β-alanine degradation and is genomically co-localized with and regulated by the orthologous gene/protein in *Sulfolobus tokodaii* [17]. Furthermore, genes encoding proteins involved in other aspects of β-alanine or coenzyme A metabolism did not display BarR association in the tested growth conditions. This indicates that there is only BarR-mediated β-alanine-dependent transcriptional regulation of the degradation of β-alanine but not of its biosynthesis.

### In vitro validation of BarR binding regions

**Fig. 2:**
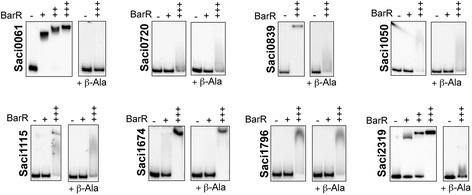
*In vitro* binding of BarR to ChIP-enriched regions. Electrophoretic mobility shift assays to test BarR binding to a set of fragments representing high-enrichment ChIP-seq targets. Targets are named according to the gene closest to or overlapping most with the ChIP-seq peak. BarR protein concentrations are indicated above each EMSA autoradiograph with: + = 0.25 μM; ++ = 1.25 μM and +++ = 2.5 μM. For each tested fragment, the left panel represents the experiment performed without addition of β-alanine, while the right panel represents the experiment performed in the presence of 1 mM β-alanine (indicated below)

The interaction between BarR protein and in vivo bound genomic regions was validated in vitro by employing EMSAs for a selection of eight high-enrichment ChIP-seq peaks (Fig. 2). In addition to the Saci2136 and Saci2137 targets, for which stable in vitro binding was demonstrated before [17], six novel targets (Saci0061, Saci0839, Saci1115, Saci1674, Saci1796, Saci2319) were shown to form stable and specific BarR-DNA complexes. BarR-DNA complexes generally displayed relative low migration velocities, indicating a higher stoichiometric nature as is the case for the previously characterized complex with the *barR*/*Saci_2137* intergenic region, in which BarR interacts with multiple regularly spaced binding sites [17]. All other tested targets displayed unstable low-affinity binding, visible by smearing. These results indicate that BarR interacts with its genomic targets in a direct and presumably sequence-specific manner.

Similarly as with the in vivo observations, the effect of β-alanine on the protein-DNA interaction varied for the different targets (Fig. 2). The complexes formed with the high-affinity targets Saci0061, Saci0839 and Saci2319 targets dissociated in response to 1 mM β-alanine, similarly as for the Saci2136 target, while for the other targets binding is only slightly or not at all affected by β-alanine.

### Identification of the BarR DNA-binding motif

**Fig. 3:**
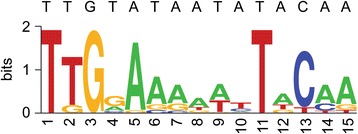
BarR DNA-binding motif. Sequence logo representing MEME predictions of the BarR DNA-binding motif. The consensus sequence for BarR based on previous binding site identifications in the *barR*/*Saci_2137* intergenic region [17], is shown above

To predict the putative binding motif in the identified BarR binding loci, ChIP-seq sequences were analyzed by MEME software, a bioinformatic tool that searches for overrepresented sequence motifs in multiple unaligned sequences [29]. This analysis resulted in the identification of a 15 bp semi-palindromic binding motif 5’-TTGGAAAAATTACAA-3’ with an E-value of 8.5e^−4^ (Fig. 3), present in 20 of 21 peaks [Additional file 3]. This predicted binding motif is congruent with the sequences of the binding sites that were previously characterized by footprinting of BarR-DNA complexes formed with the *barR*/*Saci_2137* intergenic region [17]. Together, the presence of a conserved recognition sequence and the observed in vitro binding indicates that the ChIP-enriched genomic sites are associated with BarR by direct sequence-specific interactions with the DNA and not through protein-protein interactions, as has been observed for other archaeal Lrp-type regulators [20, 32]. Notably, BarR and Ss-LrpB, an Lrp-type regulator in the related species *Sulfolobus solfataricus*, share a very similar sequence specificity [21, 33], suggesting that the helix-turn-helix motif-encoding parts of *barR* and *Ss-lrpB* share a common ancestral gene [Additional file 4].

### In vivo binding at the *barR*/*Saci_2137* genomic region

**Fig. 4:**
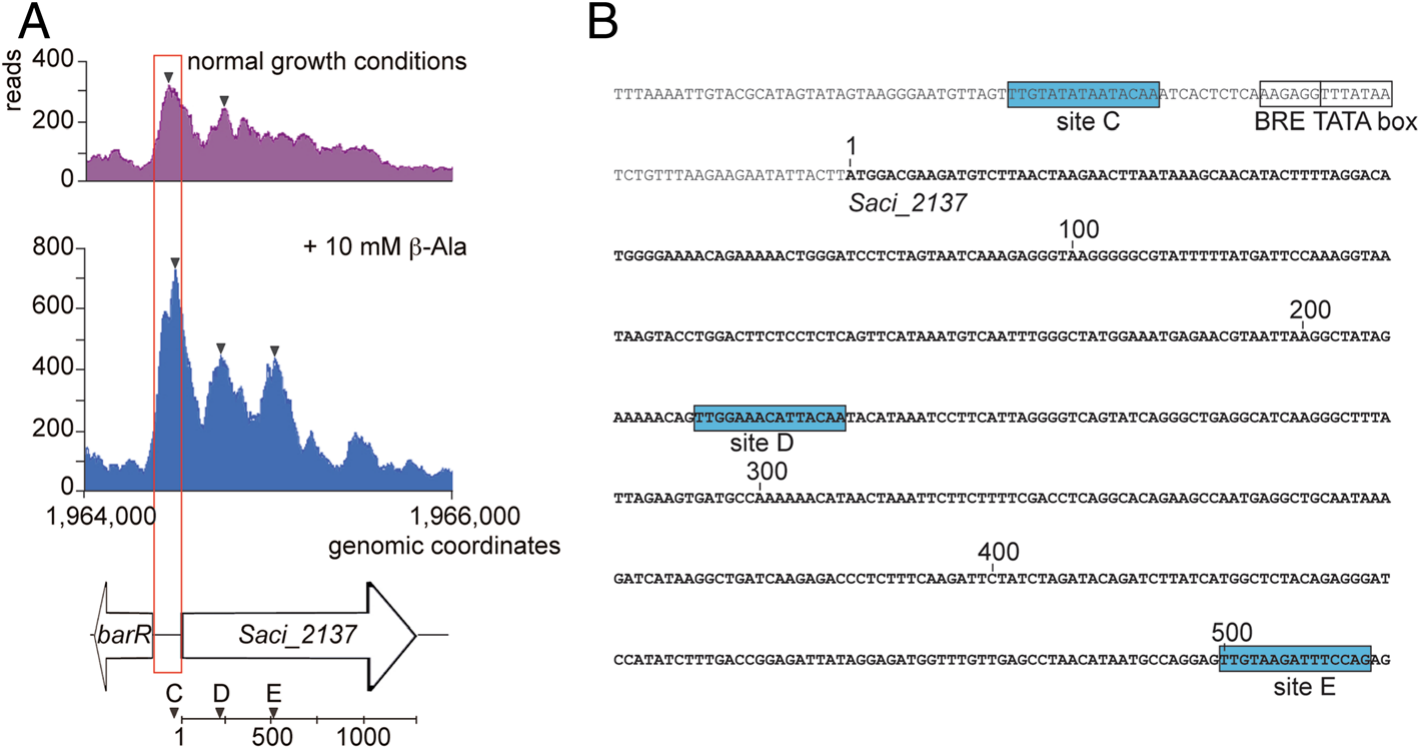
*In vivo* binding to the *barR*/*Saci_2137* genomic region. **a** Binding profile of target Saci2137 recorded in the absence (*purple*) and presence (*blue*) of β-alanine. The *barR*/*Saci_2137* intergenic region, which was previously studied, is indicated in red. Binding peaks are indicated with triangles. The position of the binding sites C, D and E with respect to the *Saci_2137* open reading frame is indicated below. **b** Sequence of the *Saci_2137* genomic region in which the regularly spaced sites C, D and E are located. Position numbering is with respect to the *Saci_2137* translation start (indicated with 1)

ChIP-seq analysis confirmed the in vivo association of BarR with the *barR*-*Saci_2137* intergenic region that is responsible for autoregulation and regulation of aminotransferase expression [17] (Fig. 4a). However, in addition to the intergenic region for which binding was characterized previously in in vitro experiments, binding extends into the coding sequence of the BarR target gene *Saci_2137*, resulting in a complex binding profile with three peak summits. *In silico* analysis of the *Saci_2137* coding sequence indeed identified the presence of two previously uncharacterized BarR binding motifs, which we named site D and site E, in addition to site C that is located upstream of the promoter (Fig. 4b). The three sites presumably mediating *Saci_2137* regulation are regularly spaced with a very similar center-to-center distance (277 bp between site C and D and 283 bp between D and E). The locations and regular spacing of the three sites is reflected by the three peak summits in the ChIP-seq profile (Fig. 4a). It can be hypothesized that binding events at the major and auxiliary sites are not taking place independently from each other. Possibly, protein-protein interactions between BarR subunits bound at these different sites, whether or not part of the same pre-existing oligomeric protein molecule, result in the formation of a higher-order nucleoprotein structure in which the intervening DNA is looped out.

Binding of BarR to the intragenic binding sites had not been detected before and might underlay the differences observed previously between in vitro and in vivo detected BarR-DNA interactions with the *barR*/*Saci_2137* intergenic region: while β-alanine causes the disruption of BarR-DNA complexes formed with a DNA fragment encompassing the intergenic region in vitro, this is not the case in vivo [17]. Furthermore, BarR activates *Saci_2137* expression in the presence of β-alanine. With regards to binding, the ChIP-seq analysis reveals an opposite effect as compared to the in vitro observations, with higher number of sequence reads when cells were grown in the presence of exogenously added β-alanine (Fig. 4a). Possibly, binding to the newly identified intragenic sites stabilizes the complex upon the conformational changes induced by β-alanine. The presence of auxiliary operator sites in the coding sequence of the regulated gene has also been observed for the Lrp-type transcription factors LysM in *S. acidocaldarius* [7] and Ss-LrpB in the related *Sulfolobus solfataricus* [6].

### Expression analysis of genes adjacent to BarR binding regions

Next, we determined whether or not BarR is involved in the regulation of the genes adjacent to the identified ChIP-seq binding regions. We performed qRT-PCR to analyze the effect of *barR* deletion on the expression of the most probable target genes, either located near high-affinity sites (*Saci_0061*, *Saci_0839*, *Saci_1674*, *Saci_1797*, *Saci_2320* and *Saci_2321*) and/or displaying a binding event in the neighbourhood of their promoter region increasing the probability that BarR binding affects gene expression (*Saci_1050*, *Saci_1797*, *Saci_2320* and *Saci_2321*). Gene expression was monitored both in the absence and presence of 10 mM β-alanine.

**Fig. 5:**
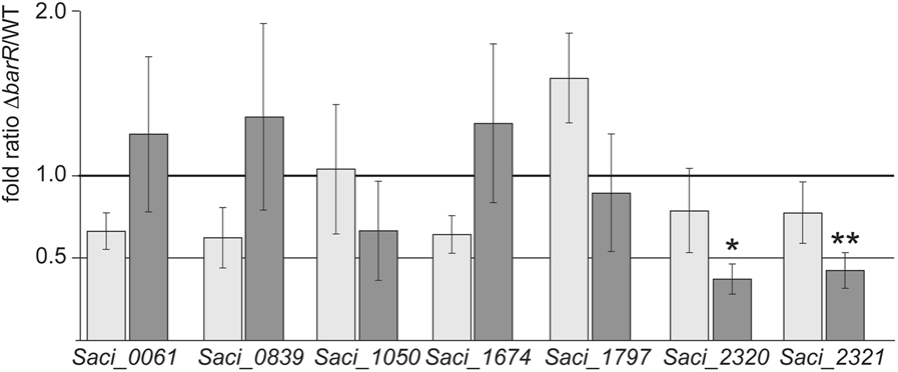
Expression analysis of genes located adjacent to BarR genomic binding sites. Relative gene expression ratios in a MW001Δ*barR* versus MW001 strain, determined by qRT-PCR analysis. Values are the average of biological quadruplicates and standard deviations represent the biological variation. An asterisk indicates a *p*-value smaller than 0.05, a double asterisk a *p*-value smaller than 0.01. All other *p*-values were larger than 0.05

Only 2 out of the 7 tested genes displayed a significantly different expression in the MW001Δ*barR* strain as compared to the isogenic MW001 (Fig. 5). These genes, *Saci_2320* and *Saci_2321,* located in an operon and encoding a glutamate synthase enzyme (*gltB*), were downregulated 2.7-fold and 2.4-fold respectively in Δ*barR* versus WT in the presence of 10 mM β-alanine. Hence, BarR also seems to activate glutamate synthase in response to β-alanine, thereby connecting the regulation of β-alanine to amino acid metabolism.

The 5 other tested genes did not show expression differences in both genetic backgrounds (Fig. 5) indicating that most sites uncovered in the ChIP-seq analysis, including intragenic sites, are non-functional in terms of transcription regulation. Given the presence of genuine BarR binding motifs and the *in vitro* validation of binding for most of these non-regulatory sites, it is unlikely that they are false positive artefacts of the ChIP-seq analysis. Also for other transcription factors, either in archaeal, bacterial or eukaryotic organisms, proof is accumulating that specific binding on the genome without an apparent regulatory output is a common feature of both global and local regulators [6, 7, 34–44]. Generally, more than half of all binding sites discovered by ChIP-seq for a transcription factor under study are intragenic and not directly linked to transcription regulation [6, 7, 35, 41–44]. The exact function of these sites, sometimes termed “decoy sites” [45] or “transcriptionally dormant sites” [40], is unclear although it is assumed that they optimize regulatory response dosage kinetics and dynamics by causing transcription factor titration and buffering [45–47]. Alternatively, these sites potentially contribute to gene regulation by regulating spurious intragenic transcription initiation events that are undetected, or by establishing long-range regulatory interactions [43]. We therefore hypothesize that most of the newly discovered BarR genomic binding sites in this study also serve an alternative function to direct transcription regulation.

### Glutamate synthase is a regulatory junction for Lrp-type regulators in *Sulfolobus* spp

**Fig. 6:**
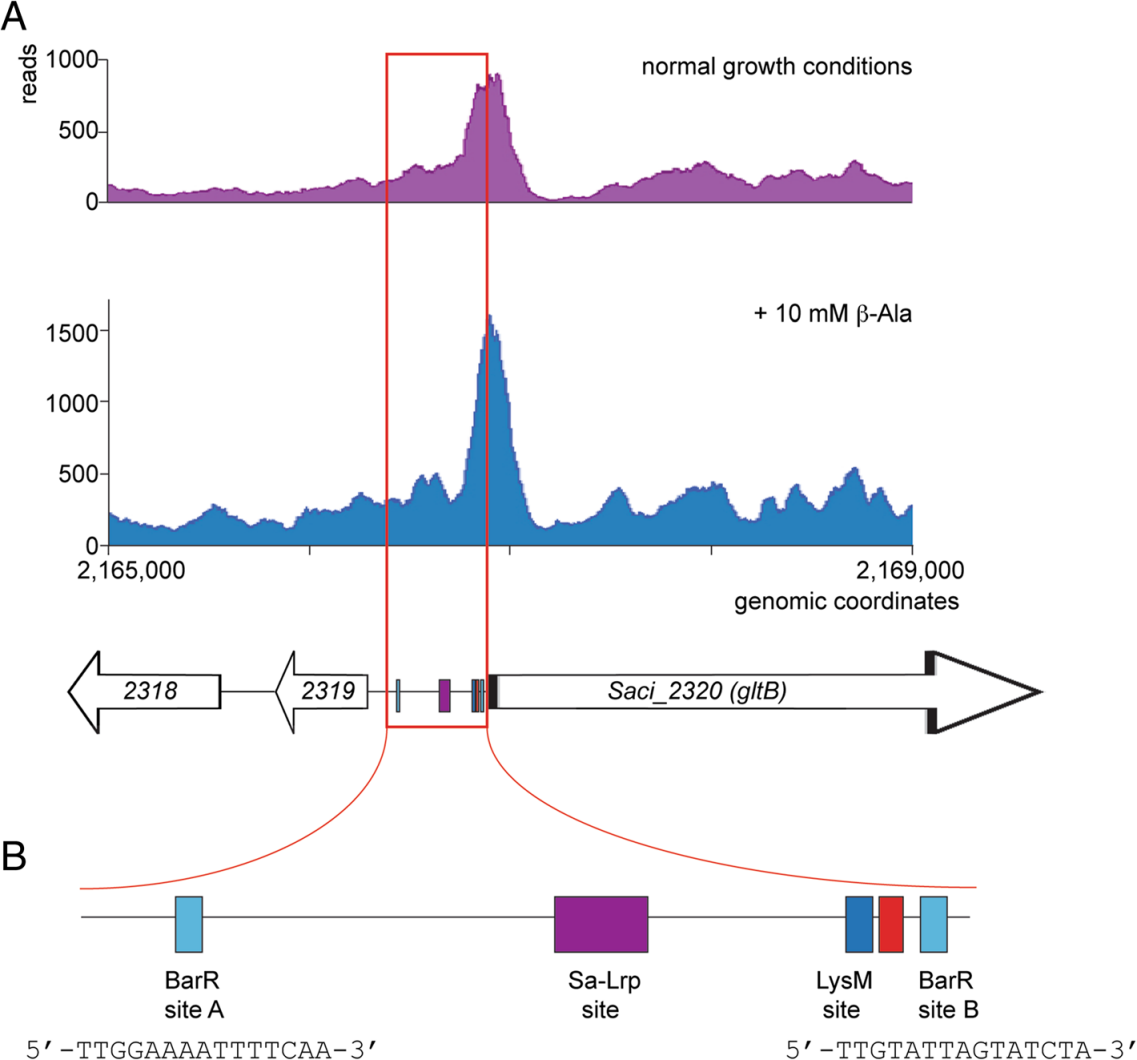
*In vivo* binding to the *gltB* promoter region. **a** Binding profile of target Saci2320 recorded in the absence (*purple*) and presence (*blue*) of β-alanine. The 500-bp intergenic region preceding the *Saci_2320* (*gltB*) open reading frame is indicated in red. Promoter and regulatory sequence elements are indicated according to the colour code shown in panel B. **b** Schematic representation of the organization of the promoter and Lrp operator elements in the *gltB* promoter/operator region. The putative 14-bp BRE/TATA box region is indicated with a red box. Sequences of the (putative) BarR sites are given below each site

The highest-enrichment ChIP-seq peak (Saci2319 target) identified in this study (Fig. 6a) is one of the few binding events that results in transcriptional activation (Fig. 5) and can be considered as the only regulatory target besides the β-alanine aminotransferase. Interestingly, the regulated *Saci_2320/Saci_2321* operon has previously been identified as a target of other Lrp-like transcription factors in *Sulfolobus* spp. (Fig. 6b). Indeed, the promoter region of glutamate synthase (*gltB*) encoded by the *Saci_2320*/*Saci_2321* is a major binding target of the glutamine-responsive non-specific binding protein Sa-Lrp [48]. In the related *S. solfataricus*, the *gltB* promoter is associated with the lysine-responsive LysM [7] through direct protein-DNA interactions at a binding site that is conserved in *S. acidocaldarius*, which harbours a LysM ortholog, directly upstream of the promoter (Fig. 6b). Furthermore, another Lrp protein Ss-LrpB also associates at the *S. solfataricus gltB* promoter through protein-protein interactions with LysM [6]. Curiously, while *S. acidocaldarius* does not contain an Ss-LrpB ortholog, the *S. acidocaldarius gltB* promoter displays at least two BarR binding sites that are very similar to the Ss-LrpB consensus sequence [Additional file 4]. This suggests a common ancestral origin of these regulatory interactions. In contrast to BarR, for none of the above-mentioned Lrp-like regulators, a clear regulatory effect on *gltB* expression was observed in single deletion strains or when comparing different relevant growth conditions [6, 7, 48]. This observation demonstrates that there is a complex interplay between the different regulators and that regulatory effects are interdependent.

The binding motif that was predicted to be recognized by BarR [Additional file 3] is located quite far upstream of the *gltB* promoter as it starts at 433 bp upstream of the *Saci_2320* translational start (Site A; Fig. 6b). While this binding motif is predicted to be a high-affinity site [Additional file 3] and is bound with high affinity *in vitro* (Fig. 2), the zoomed ChIP-seq profile displays only low-enrichment precipitation of this region (Fig. 6a). In contrast, high-enrichment precipitation is observed in the region more downstream with respect to this high-affinity site. Indeed, further *in silico* analysis enables the prediction of another BarR binding motif, not predicted by MEME, located just downstream of the promoter (Site B, Fig. 6b). Similar as for the *barR*/*Saci_2137* target, it appears that BarR binding is more complicated and consists of several operator sites. The presence of other Lrp-like regulators in the control region might explain the discrepancy between the *in vivo* observed binding profile and the theoretically predicted and *in vitro* validated binding behaviour. Of note, the predicted LysM binding site is located just upstream of the promoter, a canonical position for transcriptional activators, while the BarR site B, which is presumably bound *in vivo* and responsible for activation, exerts BarR-mediated activation.

It is interesting to note that the *Saci_2320*/*Saci_2321* operon encoding glutamate synthase is a common target of BarR and several other Lrp-family regulators in *Sulfolobus spp.* and that its control region can thus be considered as a DNA-binding hotspot for Lrp proteins. This adds to the variety of mechanisms in which archaeal Lrp-like regulators form transcription regulatory networks: i) different Lrp-like regulators bind to adjacent binding sites in the same control region (shown in this work); ii) they interact through protein-protein interactions resulting in genomic co-association [6, 20, 32], iii) paralogs share the same DNA-binding motifs [8], and iv) they regulate each other’s expression [19, 48]. For example, the transcription of *barR* has been shown to be regulated by Sa-Lrp [48].

## Conclusions

In conclusion, the ChIP-seq analysis presented in this work provides useful insights into the functioning and physiological role of BarR. We provide proof that BarR is a dedicated and mainly local acting transcriptional activator and that it has a limited regulon composed of its own gene, the *Saci_2137* aminotransferase and glutamine synthase. Besides its local role in the regulation of β-alanine degradation, we also demonstrate that BarR displays an overlapping regulon with other Lrp-like regulators by sharing glutamine synthase as a target. This adds to the growing body of evidence that Lrp-like regulators have connected functions and that the Lrp family is an important TF family for archaeal physiology.

## Abbreviations

ChIP, chromatin immunoprecipitation; ChIP-seq, chromatin immunoprecipitation combined with high-throughput sequencing; CoA, coenzyme A; EMSA, electrophoretic mobility shift assay; FFRP, feast/famine regulatory protein; gltB, glutamine synthase; HTH, helix-turn-helix; Lrp, leucine-responsive regulatory protein; OD, optical density; RAM, regulation of amino acid metabolism; RT-qPCR, reverse transcriptase quantitative polymerase chain reaction

## Additional files

Additional file 1: Temperature evolution in *S. acidocaldarius* cultures during crosslinking. (PDF 196 kb) Additional file 2: Sequences of oligonucleotides used in this work. (PDF 68 kb) Additional file 3: Predicted binding motifs for ChIP-seq enriched BarR targets. (PDF 70 kb) Additional file 4: The DNA-binding domains of S. acidocaldarius BarR and *S. solfataricus* Ss-LrpB might share a common ancestor. (PDF 198 kb)
