# Supplementary material for: The genome-scale DNA-binding profile of BarR, a β-alanine responsive transcription factor in the archaeon Sulfolobus acidocaldarius
Source: BMC Genomics. 2016 Aug 8;17:569. doi: 10.1186/s12864-016-2890-0 (PMC4977709; doi:10.1186/s12864-016-2890-0)
Supplement: Additional file 1: — Temperature evolution in S. acidocaldarius cultures during crosslinking. (PDF 196 kb) [file 12864_2016_2890_MOESM1_ESM.pdf]

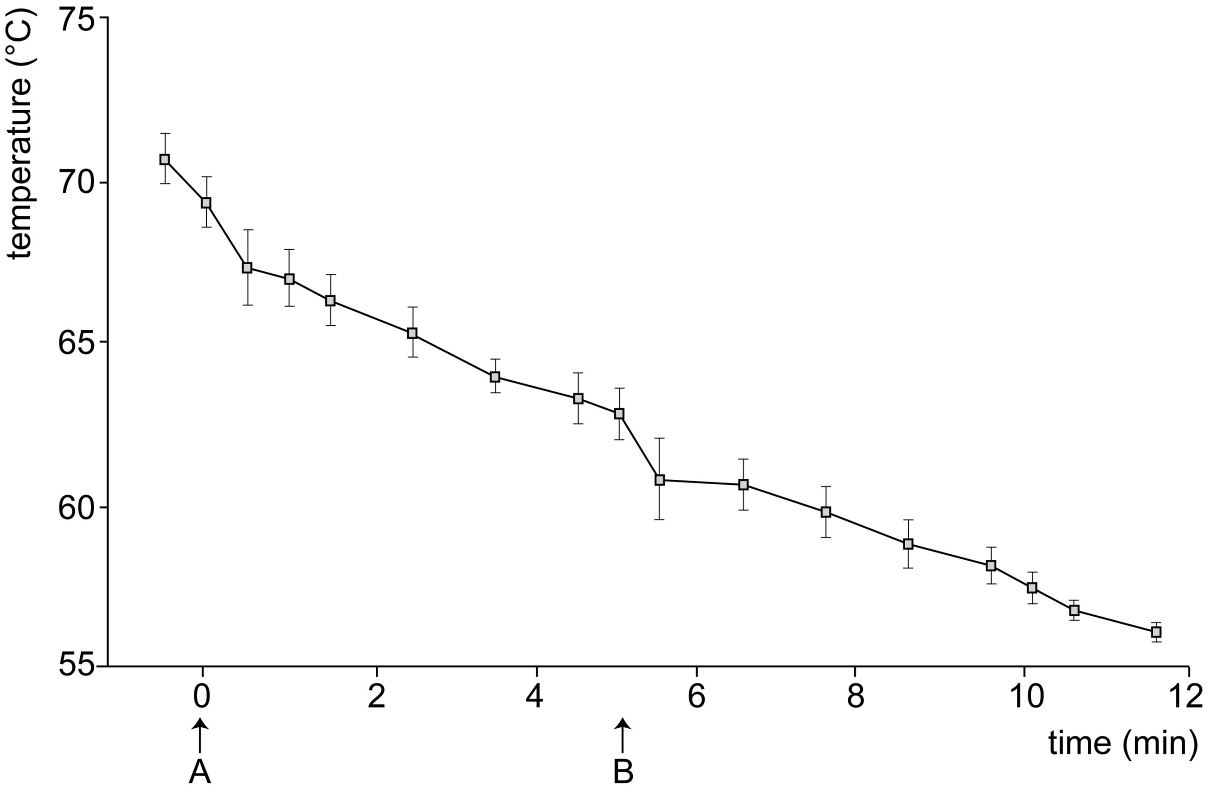

Temperature evolution in *S. acidocaldarius* cultures during crosslinking. At time point A, formaldehyde is added to the cultures and the cultures are shifted from 75°C to room temperature, while they are continuously being shaken. At time point B, glycine is added to quench the crosslinking reaction.
