# Supplementary material for: The genome-scale DNA-binding profile of BarR, a β-alanine responsive transcription factor in the archaeon Sulfolobus acidocaldarius
Source: BMC Genomics. 2016 Aug 8;17:569. doi: 10.1186/s12864-016-2890-0 (PMC4977709; doi:10.1186/s12864-016-2890-0)
Supplement: Additional file 2: — Sequences of oligonucleotides used in this work. (PDF 68 kb) [file 12864_2016_2890_MOESM2_ESM.pdf]

Additional file 2. Sequences of oligonucleotides used in this work

Primer name	Sequence	Purpose
HL009	5’-GAGGTTCTTGATAGGTAATAGAAAGTTTG-3’	Forward EMSA probe Saci0061 target
HL010	5’-CTAATGTTTTAATTGAGTCTTATAATGACGCC-3’	Reverse EMSA probe Saci0061 target
HL013	5’-GGAAAGGAAAATGAATTAGTTTTATACAGC-3’	Forward EMSA probe Saci0720 target
HL014	5’-GAGACCCAAACGCTGTTCATCATTATATC-3’	Reverse EMSA probe Saci0720 target
HL015	5’-CTTAGTAATCAAAATGTACTGAAGAG-3’	Forward EMSA probe Saci0839 target
HL016	5’-CCATAAGAACTTTATCACCTAGCAC-3’	Reverse EMSA probe Saci0839 target
HL017	5’-GCGATAGTATTTTCACAGGGTAAGAGC-3’	Forward EMSA probe Saci1050 target

HL018	5'-CTTTCTTCAAAATGTTCTTCTCCATCGGC-3'	Reverse EMSA probe Saci1050 target
HL019	5'-GAGGGCTTAAGAGAGCTAATGTAAAGC-3'	Forward EMSA probe Saci1115 target
HL020	5'-GCCCCGGCATGTCCAATTAACTCAAC-3'	Reverse EMSA probe Saci1115 target
HL021	5'-ATGAATGAGGTGATAAACTAATGATCAGG-3'	Forward EMSA probe Saci1674 target
HL022	5'-GGTCAAGTACAAATAGACCACCTGTAGG-3'	Reverse EMSA probe Saci1674 target
HL023	5'-CAAGTGCCATTAAATTAAATGTTATACAAAAC-3'	Forward EMSA probe Saci1796 target
HL024	5'-CACCTAGCTGAAAAGTCCATCATCCTC-3'	Reverse EMSA probe Saci1796 target
HL027	5'-CAACACTGATATACCAAGGATAAG-3'	Forward EMSA probe Saci2319 target
HL028	5'-GCTATTTAAATCTCAACCCCTTAGGAATG-3'	Reverse EMSA

		probe Saci2319 target
HL037	5’-TGCAGGACAAACGGATACAG-3’	Forward qRT-PCR primer *Saci_0061*
HL038	5’-AGGGCTTGTATGTCATCTCCAG-3’	Reverse qRT-PCR primer *Saci_0061*
HL039	5’-GCCATAACATCGACATCAGC-3’	Forward qRT-PCR primer *Saci_0839*
HL040	5’-CCGTAATGAGTTGCAGAACC-3’	Reverse qRT-PCR primer *Saci_0839*
HL1105	5’-TTATGGCGACGGTGAAAGAG-3’	Forward qRT-PCR primer *Saci_1050*
HL1106	5’-CCAGATGCTGAGCTTTTCC-3’	Reverse qRT-PCR primer *Saci_1050*
HL043	5’-AGGAGCGGTCTTGAGAATTG-3’	Forward qRT-PCR primer *Saci_1674*
HL044	5’-CCCACCAAAGATGACTGAAG-3’	Reverse qRT-PCR primer

		Saci_1674
HL045	5'-AAAGGGAAGGCAGTGGAAAG-3'	Forward qRT-PCR primer *Saci_1797*
HL046	5'-GCCCTTCTTGAAGCATTTTC-3'	Reverse qRT-PCR primer *Saci_1797*
HL047	5'-TTAAGGCACAGGGAATGAGG-3'	Forward qRT-PCR primer *Saci_2320*
HL048	5'-TGGCTGCATCTGTAGCATTC-3'	Reverse qRT-PCR primer *Saci_2320*
HL1115	5'-CGTAACCAAAGCCAGAAAGG-3'	Forward qRT-PCR primer *Saci_2321*
HL1116	5'-TCCTTTGAACACGCTCAGTG-3'	Reverse qRT-PCR primer *Saci_2321*
