# Supplementary material for: The genome-scale DNA-binding profile of BarR, a β-alanine responsive transcription factor in the archaeon Sulfolobus acidocaldarius
Source: BMC Genomics. 2016 Aug 8;17:569. doi: 10.1186/s12864-016-2890-0 (PMC4977709; doi:10.1186/s12864-016-2890-0)
Supplement: Additional file 3: — Predicted binding motifs for ChIP-seq enriched BarR targets. (PDF 70 kb) [file 12864_2016_2890_MOESM3_ESM.pdf]

Additional file 3. Predicted binding motifs for ChIP-seq enriched BarR targets

Target name	Strand	Start	*p*-value	Sites
Saci0061	-	46671	3E-05	TTGAGAAATATACAA
Saci0695	+	554789	9E-06	TTGCAGATATTGCAA
Saci0720	+	576907	3E-06	TTGGAAATATTGCTA
Saci0839	-	675026	5E-06	TGGGAGATTCTACAA
Saci0839	+	676093	1E-04	TTGAAAGATGTTCTT
Saci1050	+	850913	2E-07	TTGGAAAGTCTACAA
Saci1115	-	918508	7E-07	TTGGAAAATTTTCAG
Saci1182	+	999178	4E-08	TTGGAAAAACTGCAA
Saci1664	+	1421792	7E-07	TTGGAACAATTTCAA
Saci1674	-	1431617	1E-06	TTGGAAATTTTACAG
Saci1796	-	1563358	3E-05	TTGTAAAAACTACCT
Saci1796	+	1564045	5E-05	TTGAACCGAGTACCA
Saci1833	-	1598811	4E-07	TTGGAAAAAATACAA
Saci1964	+	1773962	4E-06	TTGCAGGAACTACAA
Saci2025	+	1840076	5E-06	TTGAACAAATTGCCA
Saci2073	-	1890084	9E-05	TGGGAAGAGTTATCG
Saci2136	-	1964107	8E-06	TGGAAAGGATTACCA
Saci2137	+	1964732	5E-07	TTGGAAACATTACAA
Saci2166	+	2000254	9E-06	TTGGAAGATTTTTAA
Saci2319	+	2166440	1E-07	TTGGAAAATTTTCAA
