# Supplementary material for: The genome-scale DNA-binding profile of BarR, a β-alanine responsive transcription factor in the archaeon Sulfolobus acidocaldarius
Source: BMC Genomics. 2016 Aug 8;17:569. doi: 10.1186/s12864-016-2890-0 (PMC4977709; doi:10.1186/s12864-016-2890-0)
Supplement: Additional file 4: — The DNA-binding domains of S. acidocaldarius BarR and S. solfataricus Ss-LrpB might share a common ancestor. (PDF 198 kb) [file 12864_2016_2890_MOESM4_ESM.pdf]

A

```
BarR     5′-T T G G A A A A A T T A C A A-3′
Ss-LrpB  5′-T T G C A A A A T T T G C A A-3′
```

B

```
                           α1                 α2            α3          β1
BarR     MYYIQ-MELDEIDIKILKILQENAKQSLEDMSEMLKLPKSTIAYRIKRLESQGIIKGYYAHIDP
Ss-LrpB  MMGVNIIRLDDTDEKILNILRYNAKKSLKELSDELGIPISTVRYRIKRLEDAQIIRGYVALIDR
```

The DNA-binding domains of *S. acidocaldarius* BarR and *S. solfataricus* Ss-LrpB might share a common ancestor. **A**. Alignment of the BarR and Ss-LrpB binding motifs. **B**. Amino acid sequence alignment of the DNA-binding domains of BarR and Ss-LrpB, with indication of the predicted secondary structure elements. Conserved residues are indicated in bold. The sequence identity for both domains is 61%, which is higher than the average sequence identities for Lrp-like transcription regulators, and is especially high in the recognition helix α3 and in the loop connecting α2 and α3. These regions are implicated in establishing sequence-specific contacts with the major groove of the DNA.
